# Stress-reducing effect of laughter in live comedy performance from salivary α-amylase and salivary oxytocin

**DOI:** 10.1186/s13104-025-07505-8

**Published:** 2025-10-28

**Authors:** Kayo Horie, Naoki Nanashima, Naoya In, Toshiko Tomisawa

**Affiliations:** 1 Department of Bioscience and Laboratory Medicine, Hirosaki University Graduate School of Health Sciences, Hirosaki, Japan; 2 Department of Nutrition, Faculty of Health Sciences, Aomori University of Health and Welfare, Aomori, Japan; 3 Department of Nursing Sciences, Hirosaki University Graduate School of Health Sciences, Hirosaki, Japan

**Keywords:** Laughter, Live comedy performance, Salivary α-amylase, Salivary oxytocin, Psychological stress indicators

## Abstract

**Objective:**

Laughter is recognized for its potential to alleviate stress. Salivary α-amylase (sAA) is a well-known stress marker; however, the relationship between salivary oxytocin (sOXT) and stress reducing effects by laughter remains unclear. We previously determined the relationship between laughter and optimism, anxiety, and amylase; In this study, the relationship between spontaneous laughter and stress reduction during a live comedy performance (LCP) was investigated by measuring sAA and sOXT activity before and after the performance.

**Results:**

Pre-LCP sAA was significantly positively correlated with a decreasing post-sAA/pre-sAA ratio; the scale of this effect was age dependent. Furthermore, participants who reported laughing ‘several times a month’ showed the highest value in the post-/pre-LCP sAA ratio. In contrast, those who reported laughing ‘many times a day’ had the lowest value. Contrary to our expectations, sOXT levels were significantly lower in post-LCP samples than they were in pre-LCP samples. This is thought to be because OXT has an anti-stress hormone effect, and stress was reduced after LCP, resulting in a decrease in oxytocin secretion. Our findings offer new insight into the stress-reducing effects of laughter.

**Supplementary Information:**

The online version contains supplementary material available at 10.1186/s13104-025-07505-8.

## Introduction

Recent studies have reported the positive role of laughter in enhancing quality of life [1–3], and low- or no-laughter habits have been linked to a higher risk of developing stress [4]. Furthermore, spontaneous laughter is associated with a greater reduction in cortisol levels, a stress marker, compared with other usual activities, both spontaneous and auditory laughter may achieve a relaxing effect by increasing the activity of the parasympathetic nervous system [5]. Numerous studies have reported the health benefits of laughter; as such, therapeutic laughter can be considered a non-invasive, cost-effective, and easily implemented intervention [3, 6]. In our previous study, we showed that attending a 2-h live comedy performance (LCP) increased optimism and decreased stress level, pessimism, and anxiety; these effects were more pronounced in those who laughed regularly in their everyday lives [7]. In this study, LCP participants’ sAA levels were used as a stress marker to further investigate the effects of laughter intervention with respect to age and frequency of laughter. In addition, the relationship between spontaneous laughter and OXT, which are currently not well understood, were investigated.

Regarding stress evaluation, blood sampling is invasive and can cause stress; thus, saliva is used as an alternative [8]. Salivary α-amylase activity (sAA) exhibits a faster response to stress than does salivary cortisol, and is an important biomarker that can detect changes in sympathetic nervous system activity [9–11]. Moreover, it is a good indicator of acute mental stress, and many studies have exploited this property as a rapid response to direct neural activity [12–15].

Several reports have also addressed the relationship between chronic stress and sAA [16, 17]. Although many reports have examined laughter and stress using saliva IgA and cortisol samples [18–22], only a few have investigated the relationship between sAA and laughter [23, 24].

Oxytocin (OXT) release plays an essential role during and after childbirth [25, 26], and has been called ‘the love hormone’ [27]. Furthermore, OXT modulates several other social and physiological functions such as social recognition, trust building, anti-nociception, anti-inflammation, stress relief, and feeding suppression [26]. OXT neurons are activated by various stress stimuli [28], and its release can influence the autonomic nervous and immune systems, serving as a stress-coping, anti-inflammatory, and antioxidant molecule [29]. Furthermore, OXT has been reported to have anti-stress effects in animal studies and clinical reports in humans [30, 31]. Recently, saliva samples have been recognized as a useful noninvasive source for the measurement of OXT levels [32, 33].

Considering all of the above, we hypothesized that stress would decrease after LPC, resulting in a decrease in sAA, a stress factor, and an increase in sOXT, a ‘happy hormone’.To test this hypothesis, we investigated the relationship between spontaneous laughter and stress reduction during an LCP by measuring salivary sAA and sOXT activity before and after the performance.

## Materials and methods

### Study design and setting

Following our previous study [7], a pre–post design was used to evaluate the effects of laughter intervention induced by an LCP conducted at Hirosaki City Hall on September 10, 2023; the LCP was from 6 to 7 pm. Participants were asked to arrive early to the venue to collect salivary samples before the LCP (pre-LCP). A second salivary sample was then collected following the LCP (post-LCP). Each participant received an honorarium after all pre- and post-LCP research data were collected.

### Live comedy performance

Four famous Japanese comedians performed their routines during a 2-h LCP organized by the Division of Cultural Promotion in Hirosaki City to support citizens’ health through comedic entertainment as a form of culture.

### Participants

Participants included 104 healthy male and female individuals aged 18–64 years who attended the LCP; self-reported participant characteristics (collected before the LCP) are listed in Supplementary Table S1. All 104 samples were subjected to sAA analysis; in addition, 59 samples were also used for OXT analysis (sOXT) after obtaining additional consent.

### Salivary sample collection

Salivary samples were collected before and after the LCP; participants were asked not to eat or drink for 1 h before the experiment. Saliva was collected using the passive drool method using a saliva collection aid (Funakoshi, Tokyo, Japan). The samples were immediately stored on ice, and subsequently stored at − 20˚C until analysis.

### Measurement of sAA and sOXT levels

Of the 104 samples, 2 were excluded because of abnormally high values; thus, 102 samples were used for sAA analysis. sAA was measured using a salivary α-amylase kinetic enzyme assay kit (Funakoshi, Tokyo, Japan) according to the manufacturer’s protocol. Briefly, on the day of the assay, the saliva samples were completely thawed, vortexed, and centrifuged at 1500×*g* for 15 min. Subsequently, a 1:200 dilution was achieved in two serial steps. For the assay, 8 µL of diluted samples were added to individual wells in a 37 ℃ preheated multi-well plate, and 320 µL of the 37 ℃ preheated substrate was added to each well. Then, the optical density at 405 nm and 37 °C was measured at exactly 1 and 3 min using a Multimode Microplate Readers TriStar LB 941 (Berthold Japan, Tokyo, Japan). Enzyme activity was calculated by subtracting the 1-min reading from the 3-min reading, and multiplying the result by the conversion factor. The rate or ratio of decrease in sAA level following the LCP intervention was calculated as follows: (pre-LCP sAA − post-LCP sAA)/pre-LCP sAA × 100.

The sOXT level was measured using an Oxytocin ELISA kit (CosmoBio, Tokyo, Japan) according to the manufacturer’s protocol. On the day of the assay, saliva samples were completely thawed, vortexed, and centrifuged at 1500×*g* for 15 min. Next, the supernatant was collected, and its volume was determined. The samples were then completely dried using a vacuum concentrator system (Thermo Fisher Scientific K.K., Tokyo, Japan), and 250 µL of assay buffer was added. The samples were resuspended and completely dissolved in an ultrasonic homogenizer (Taitec, Saitama, Japan). Standard diluent or samples (100 µL) were added to each well of a multi-well plate and incubated overnight at 4 °C. All standards and samples were analyzed in duplicate. Next, after washing each well, 200 µL of substrate was added and incubated for 60 min at room temperature. Next, each well was washed again and 50 µL stop solution was added; the optical density at 405 nm was measured using a microplate reader (Bio-Rad Laboratories, Tokyo, Japan).

### Statistical analysis

Statistical analyses were conducted using BellCurve for Excel version 4.06 (Social Survey Research Information, Tokyo, Japan). Post sAA did not follow a normal distribution, with a Shapitro–Wilk test of *p* < 0.001, and so non-parametric tests were performed. Mann–Whitney *U* tests were used to compare two groups; Kruskal–Wallis analysis with the Steel post-hoc test was used for multi-group comparisons. The Spearman’s rank correlation coefficient was employed to assess the correlation between pre-LCP sAA and the post-/pre-LCP sAA ratio; *p* < 0.05 was considered statistically significant.

## Results

### Salivary α-amylase

**Fig. 1:**
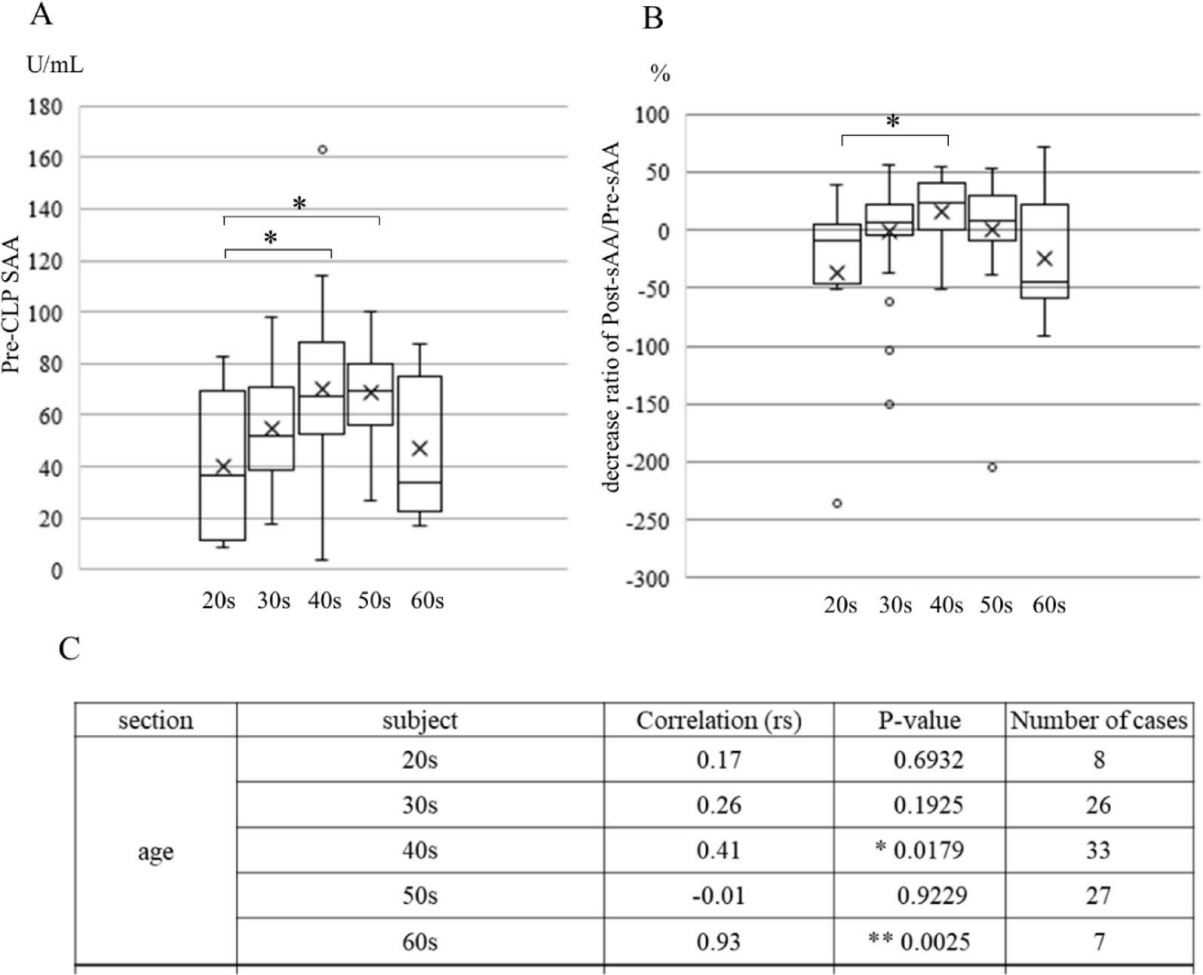
Pre-CLP-sAA and post/pre-sAA decrease ratio. **A** Pre-CLP-SAA by age; **B** decrease ratio of post-sAA/pre-sAA by age. Statistical analysis: Kruskal–Wallis analysis with the Steel post hoc test. *p < 0.05. **C** Correlation between the post-sAA/pre-sAA decrease ratio and pre-CLP-SAA by age or frequency of laughter, determined using Spearman’s rank correlation. *p* < 0.05, **p < 0.01

Our previous study showed that the overall sAA level was significantly lower post-LCP than it was pre-LCP intervention [7]. In this study, we determined the pre-LCP sAA and decreases in the post-/pre-LCP sAA ratio according to age (Fig. 1). Only 1 participant was aged < 18 years’ therefore, this group was excluded from the analysis because of the small number of cases (Fig. 1A). Pre-LCP sAA levels were significantly higher in participants in their 40 s and 50 s than they were in those in their 20 s (*p* = 0.036 and *p* = 0.024, respectively). Similarly, the decrease in the post-/pre-LCP sAA ratio was significantly higher in participants in their 40 s than it was for those in their 20 s (*p* = 0.017; Fig. 1B). As shown in Fig. 1C, pre-LCP sAA was significantly positively correlated with decreasing post-sAA/pre-sAA ratio based on a Spearman’s rank correlation test in participants in their 40 s (rs = 0.41; *p* = 0.0178) and 60 s (rs = 0.93; *p* = 0.003).

**Fig. 2:**
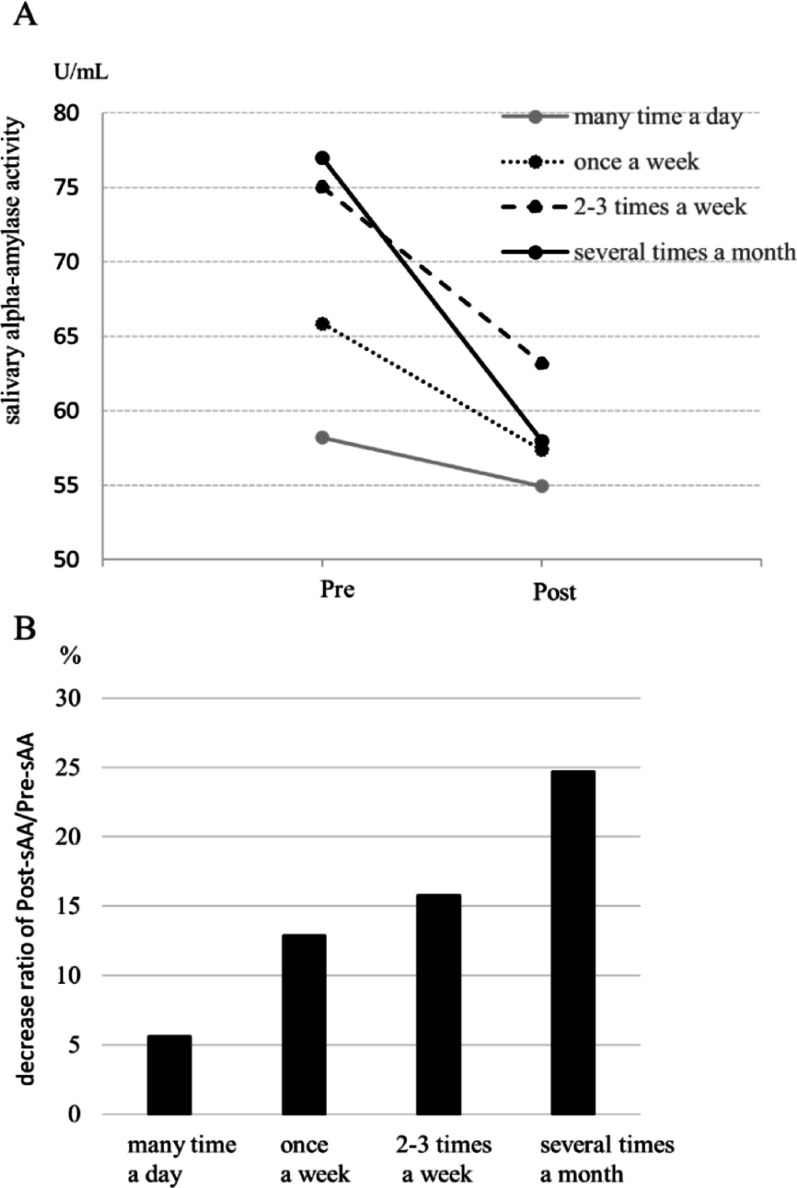
Post/pre-SAA decrease ratio in frequency of laughter. **A** Pre- and post-CLP-sAA in frequency of laughter; **B** decrease ratio of post-sAA/pre-sAA by frequency of laughter, U/mL; units per milliliter, *sAA* salivary α-amylase activity

Figure 2 show changes in sAA levels before and after the LCP according to the self-reported frequency of laughter. No significant differences emerged, but pre-LCP sAA tended to increase as frequency of laughter decreased. Similarly, the post-/pre-LCP sAA ratio tended to increase as the frequency of laughter decreased. Those who reported laughing ‘several times a month’ showed the highest pre-LCP sAA and greatest decrease in post-/pre-LCP sAA ratio; however, those who reported laughing ‘many times a day’ showed the lowest values.

### Salivary oxytocin

**Fig. 3:**
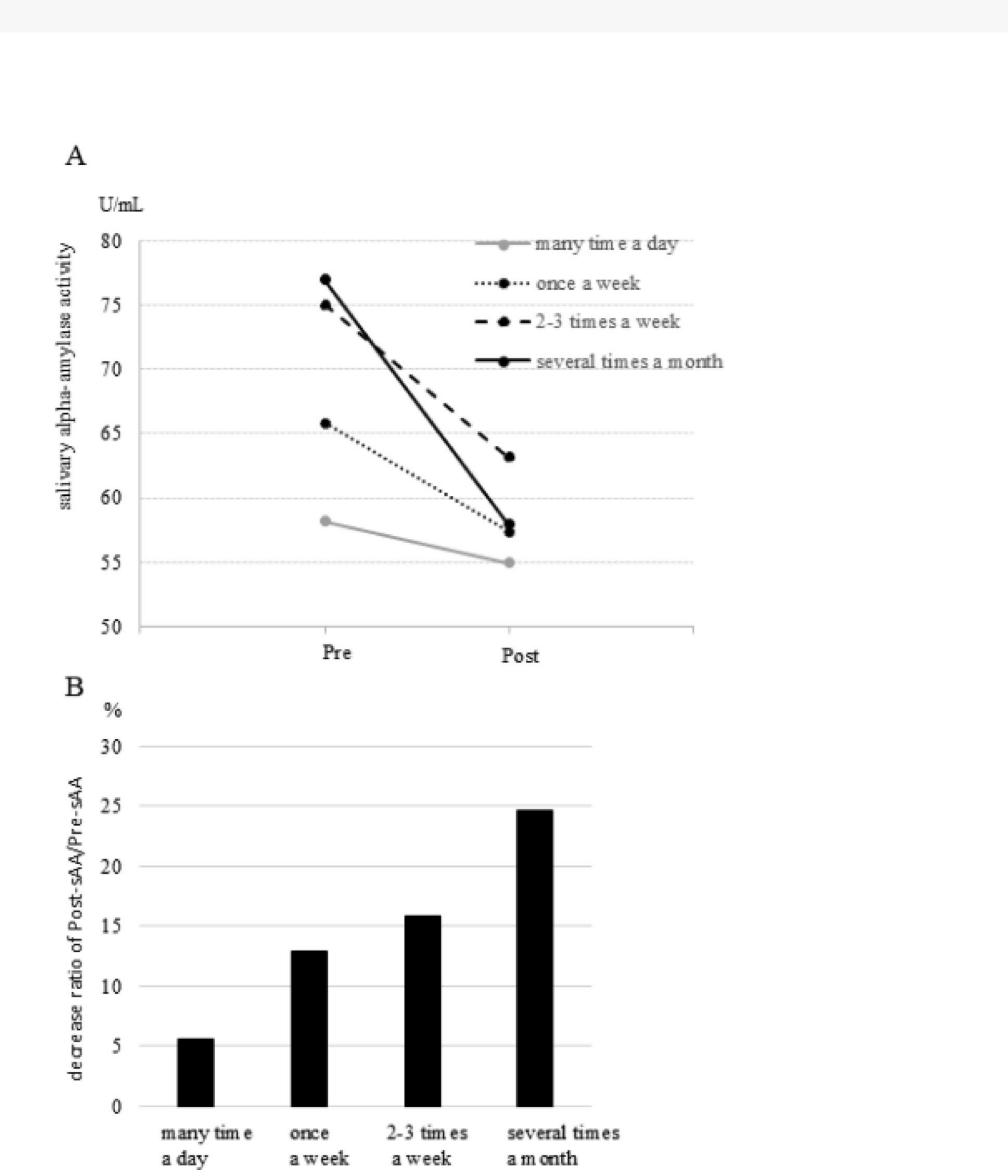
Changes in salivary oxytocin activity. **A** Pre- and post-CLP-OXT activity in females; **B** pre- and post-CLP-OXT activity in males; **C** overall pre- and post-CLP-OXT activity; **D** pre- and post-CLP-OXT activity by age. Statistical analysis: Mann–Whitney U test, *p < 0.05, * * *p* < 0.01. *CLP* comedy live performance, *SAA* salivary α-amylase activity

As shown in Fig. 3A–C, all sOXT levels were significantly lower after LCP intervention than they were before the LCP (female participants: *p* = 0.015; male participants: *p* = 0.0494; overall: *p* = 0.002). Although it showed no significant difference, in many age groups, s-OXT was reduced post-LCP compared with that pre-LCP. Furthermore, sOXT levels increased among participants in their 50 s and 60 s to a degree greater than they did in younger participants (Fig. 3D).

## Discussion

SAA has a faster response to stress than does salivary cortisol, which has long been used for stress assessment and has recently attracted attention as an objective stress assessment method [34, 35]. In this study, sAA was used as an assessment tool to evaluate stress levels; the relationship between laughter intervention in the form of an LCP and resulting stress levels of participants was examined. The study design did not induce excessive stress before the LCP intervention; therefore, pre-LCP sAA levels were considered to represent the baseline stress state.

In the present study, individuals in their 40 s and 50 s had higher pre-LCP sAA. In addition, these groups had a greater decrease in the post-/pre-LCP sAA ratio, and a positive correlation was observed between pre-LCP sAA and the decrease in post-/pre-LCP sAA ratio among participants in their 40s. These results might reflect that later life is associated with greater stress [36]. Furthermore, participants who reported laughing ‘several times a month’ showed the greatest decrease in the sAA ratio before and after LCP; we suggest that this is because those that laugh only several times a month are more likely to be in a high stress state and may have experienced the highest LCP-induced decrease in SAA owing to the LCP intervention.

No significant differences in pre-LCP and post-LCP sAA were found among groups reporting different frequencies of laughter; however, the decrease in post-/pre-LCP sAA ratios and pre-LCP sAA levels tended to increase as laughter frequency decreased. These results suggest that laughter intervention through LCP might suppress sympathetic arousal caused by chronic psychological stress and increase parasympathetic nervous system activity, producing a relaxation effect.

In recent years, OXT has become one of the most studied peptides of the human neuroendocrine system [37]. In addition, saliva is recognized as a useful sample for OXT assessment because it can be collected non-invasively [38]. Contrary to our expectations, this study did not find any increase in sOXT in the post-LCP samples. This might be because stress was reduced post-LCP resulting in a decrease in oxytocin secretion. OXT has also been shown to produce anxiolytic and anti-stress effects [39], directly attenuate decreased cortisol levels and activation of the hypothalamic–pituitary–adrenal (HPA) axis, and is associated with reduced stress-induced psychological health risks [40, 41].

Only a small number of reports have directly addressed laughter’s involvement in oxytocin regulation. Pfundmair showed that neither the self-reported effect nor the perceived funniness of a comedy video affected OXT levels [42].; that is, OXT did not affect socially irrelevant inner cheerfulness.

## Conclusion

Using sAA as an objective marker, we clarified that laughter intervention by participation in a CLP may suppress chronic psychological stress. Furthermore, we analyzed sAA and sOXT levels using the same saliva sample for the first time and found that OXT has potential as a stress marker. Further detailed analyses of other factors, such as immune response-related stress and happiness markers, at various time points, are needed. The results of this study provide new insight into the stress-reducing effects of laughter.

## Limitations

This study design did not induce excessive stress before the LCP intervention. Thus, the pre-LCP sAA levels were defined as a control. In addition, there is a lack of objective measures to evaluate humor.

We had no information regarding smoking status and medication use was unavailable. In addition, factors such as timing and seating arrangements may influence stress reduction independent of the comedy performance.

Although it was not possible in this study owing to the limited amount of saliva sample, we believe that further detailed analysis at various points of time is necessary to determine the relationship between the stress-reducing effects of OXT and LCP.

## Supplementary Information

Supplementary Material 1.

## Data Availability

All data that supports the findings of this study are available within the article and/or supplementary materials.
