# Supplementary figures and images for: Stress-reducing effect of laughter in live comedy performance from salivary α-amylase and salivary oxytocin
Source: BMC Res Notes. 2025 Oct 28;18:456. doi: 10.1186/s13104-025-07505-8 (PMC12570744; doi:10.1186/s13104-025-07505-8)

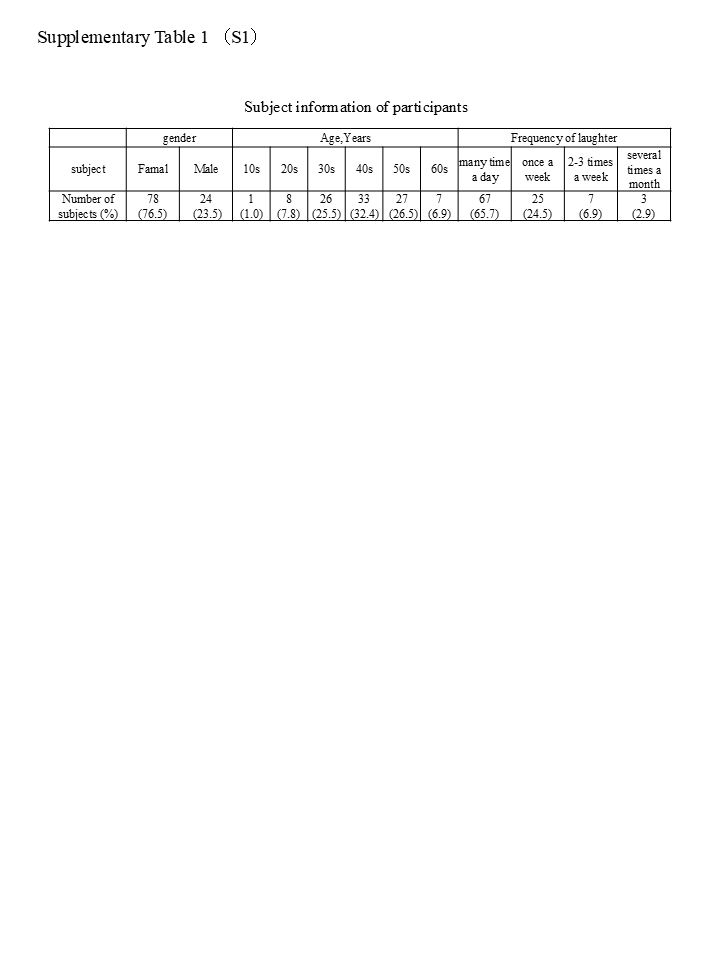

Supplement: Supplementary file 1 — Supplementary Material 1. [file 13104_2025_7505_MOESM1_ESM.tif]
